# A Simple Approach to Ranking Differentially Expressed Gene Expression Time Courses through Gaussian Process Regression

**DOI:** 10.1186/1471-2105-12-180

**Published:** 2011-05-20

**Authors:** Alfredo A Kalaitzis, Neil D Lawrence

**Affiliations:** 1 The Sheffield Institute for Translational Neuroscience, 385A Glossop Road, Sheffield, S10 2HQ, UK

## Abstract

**Background:**

The analysis of gene expression from time series underpins many biological studies. Two basic forms of analysis recur for data of this type: removing inactive (quiet) genes from the study and determining which genes are differentially expressed. Often these analysis stages are applied disregarding the fact that the data is drawn from a time series. In this paper we propose a simple model for accounting for the underlying temporal nature of the data based on a Gaussian process.

**Results:**

We review Gaussian process (GP) regression for estimating the continuous trajectories underlying in gene expression time-series. We present a simple approach which can be used to filter quiet genes, or for the case of time series in the form of expression ratios, quantify differential expression. We assess via ROC curves the rankings produced by our regression framework and compare them to a recently proposed hierarchical Bayesian model for the analysis of gene expression time-series (BATS). We compare on both simulated and experimental data showing that the proposed approach considerably outperforms the current state of the art.

**Conclusions:**

Gaussian processes offer an attractive trade-off between efficiency and usability for the analysis of microarray time series. The Gaussian process framework offers a natural way of handling biological replicates and missing values and provides confidence intervals along the estimated curves of gene expression. Therefore, we believe Gaussian processes should be a standard tool in the analysis of gene expression time series.

## Background

Gene expression profiles give a snapshot of mRNA concentration levels as encoded by the genes of an organism under given experimental conditions. Early studies of this data often focused on a single point in time which biologists assumed to be critical along the gene regulation process after the perturbation. However, the *static *nature of such experiments severely restricts the inferences that can be made about the underlying dynamical system.

With the decreasing cost of gene expression microarrays time series experiments have become commonplace giving a far broader picture of the gene regulation process. Such time series are often irregularly sampled and may involve differing numbers of replicates at each time point [1]. The experimental conditions under which gene expression measurements are taken cannot be perfectly controlled leading the signals of interest to be corrupted by noise, either of biological origin or arising through the measurement process.

Primary analysis of gene expression profiles is often dominated by methods targeted at *static *experiments, i.e. gene expression measured on a single time-point, that treat time as an additional experimental factor [1-6]. However, were possible, it would seem sensible to consider methods that can account for the special nature of time course data. Such methods can take advantage of the particular statistical constraints that are imposed on data that is naturally ordered [7-12].

The analysis of gene expression microarray time-series has been a stepping stone to important problems in systems biology such as the genome-wide identification of direct targets of transcription factors [13,14] and the full reconstruction of gene regulatory networks [15,16]. A more comprehensive review on the motivations and methods of analysis of time-course gene expression data can be found in [17].

### Testing for Expression

**Figure 1:**
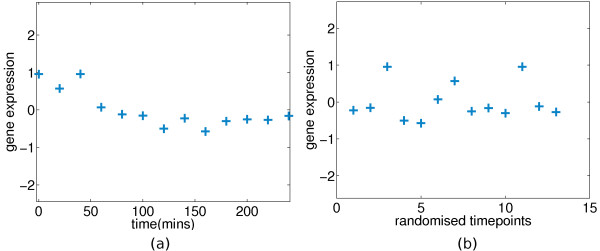
**Temporal information removed from the profile of gene Cyp1b1 in the experimental mouse data**. **(a) **The centred profile of the gene *Cyp1b1 *(probeID 1416612_at in the *GSE10562 *dataset). The blue crosses represent zero-mean hybridised gene expression in time of measurement (log2 ratios between treatment and control). **(b) **The same profile with its timepoints randomised.

A primary stage of analysis is to characterize the activity of each gene in an experiment. Removing inactive or *quiet *genes (genes which show negligible changes in mRNA concentration levels in response to treatments/perturbations) allows the focus to dwell on genes that have responded to treatment. We can consider two experimental set ups. Firstly, we may be attempting to measure the absolute level of gene expression (for example using Affymetrix GeneChip microarrays). In this case a quiet gene would be one whose expression level is indistinguishable from noise. Alternatively, we might be may be hybridizing two samples to the same array and quantifying the ratio of the expression levels. Here a quiet gene would be one which is showing a similar response in both hybridized samples. In either case we consider such expression profiles will consist principally of *noise*. Removing such genes will often have benign effects later in the processing pipeline. However, mistaken removal of profiles can clearly compromise any further downstream analysis. If the temporal nature of the data is ignored, our ability to detect such phenomena can be severely compromised. An example can be seen in Figure 1, where the temporal information is removed from an experimental profile by randomly reordering its expression samples. Disregarding the temporal correlation between measurements, hinders our ability to assess the profile due to critical inherent traits of the signal being lost such as the speed and scale of variation.

Failure to capture the signal in a profile, irrespective of the amount of embedded noise, may be partially due to *temporal aggregation *effects, meaning that the coarse sampling of gene expression or the sampling rates do not match the natural rates of change in mRNA concentrations [18]. For these reasons, the classification scheme of differential expression in this paper is focused on reaching a high *true positive rate *(TPR, *sensitivity *or *recall *) and is to serve as a pre-processing tool prior to more involved analysis of time-course microarray data. In this work we distinguish between *two-sample *testing and experiments where *control *and *treated *cases are directly-hybridized on the microarray (For brevity, we shall refer to experiments with such setups as *one-sample testing*). The *two-sample *setup is a common experimental setup in which two groups of sample replicates are used [13,19]; one being under the treatment effect of interest and the other being the control group, so to recover the most active genes under a treatment one may be interested in testing for the statistical significance of a treated profile being differentially expressed with respect to its control counterpart. Other studies use data from a *one-sample *setup [11,12], in which the *control *and *treated *cases are directly hybridized on a microarray and the measurements are normalized log fold-changes between the two output channels of the microarray [20], so the analogous goal is to test for the statistical significance of having a non-zero signal.

A recent significant contribution in regards to the estimation and ranking of differential expression of time-series in a *one-sample *setup is a hierarchical Bayesian model for the analysis of gene expression time-series (BATS) [11,12] which offers fast computations through exact equations of Bayesian inference, but makes a considerable number of prior biological assumptions to accomplish this (cf. **Simulated data**).

### Gene Expression Analysis with Gaussian Processes

*Gaussian processes*(GP) [21,22] offer an easy to implement approach to quantifying the true signal and noise embedded in a gene expression time-series, and thus allow us to rank the differential expression of the gene profile. A Gaussian process is the natural generalisation of a multivariate Gaussian distribution to a Gaussian distribution over *a specific family of functions *-- a family defined by a *covariance function *or *kernel*, i.e. a metric of similarity between data-points (Roughly speaking, if we also view a function as a vector with an infinite number of components, then that function can be represented as a point in an infinite-dimensional *space of a specific family of functions *and a Gaussian process as an infinite-dimensional Gaussian distribution over that space).

In the context of expression trajectory estimation, a Gaussian process coupled with the *squared-exponential *covariance function (or *radial basis function*, RBF) -- a standard covariance function used in regression tasks -- makes the reasonable assumption that the underlying true signal in a profile is a *smooth *function [23], i.e. a function with an infinite degree of differentiability. This property endows the GP with a large degree of flexibility in capturing the underlying signals without imposing strong modeling assumptions (e.g. number of basis functions) but may also erroneously pick up spurious patterns (false positives) should the time-course profiles suffer from temporal aggregation. From a generative viewpoint, the profiles are assumed to have been corrupted by additive white Gaussian noise. This property makes the GP an attractive tool for bootstrapping simulated biological replicates [24].

In a different context, Gaussian process priors have been used for modeling transcriptional regulation. For example in [25], while using the time-course expression of a-priori known direct targets (genes) of a transcription-factor, the authors went one step further and inferred the concentration rates of the transcription-factor protein itself and [26] extended the same model for the case of regulatory repression. The ever-lingering issue of outliers in time series is still critical, but is not addressed here as there is significant literature on this issue in the context of GP regression, which is complementary to this work.

For example [19,27] developed a probabilistic model using Gaussian processes with a robust noise model specialised for two-sample testing to detect *intervals *of differential expression, whereas the present work optionally focuses on *one-sample *testing, to rank the differential expression and ultimately detect *quiet/active *genes. Other examples can also be easily applied; [28] use a Student-*t *distribution as the robust noise model in the regression framework along with variational approximations to make inference tractable, and [29] employ a Student-*t *observation model with Laplace approximations for inference. The standard GP regression framework is straightforward to use here with a limited need for manual tweaking of a few hyper-parameters. We describe the GP framework, as used here for regression, in more detail in the **Methods **section.

## Results and Discussion

We apply standard Gaussian process (GP) regression and the Bayesian hierarchical model for the analysis of time-series (BATS) on two in-silico datasets simulated by BATS and GPs, and on one experimental dataset coming from a study on primary mouse keratinocytes with an induced activation of the TRP63 transcription factor, for which a reverse-engineering algorithm was developed (TSNI: time-series network identification) to infer the direct targets of TRP63 [13].

We assume that each gene expression profile can be categorized as either quiet or differentially expressed. We consider algorithms that provide a rank ordering of the profiles according to which is most likely to be non-quiet (or differentially expressed). Given ground truth we can then evaluate the quality of such a ranking and compare different algorithms. We make use of *receiver operating characteristic *curves (ROC curves) to evaluate the algorithms. These curves plot the *false positive rate *on the horizontal axis, versus the *true positive rate *on the vertical axis; i.e. the percentage of the total negatives (non-differentially expressed profiles) erroneously classified as positives (differentially expressed) versus the percentage of the total positives correctly classified as positives.

From the output of each model a ranking of differential expression is produced and assessed with ROC curves to quantify how well in accordance to each of the three ground truths (BATS-sampled, GP-sampled, TSNI-experimental) the method performs. The BATS model can employ three different noise models, where the marginal distribution of the error is assumed to be either Gaussian, Student-*t *or double exponential respectively. For the following comparisons we plot four ROC curves, one for each noise model of BATS and one for the GP. We demonstrate that the ranking of the GP framework outperforms that of BATS with respect to the TSNI ranking on the experimental data and on GP-sampled profiles.

### Simulated data

The first set of in-silico profiles are simulated by the BATS software http://www.na.iac.cnr.it/bats/ in accordance to the guidelines given in [12]. In BATS [11] each time-course profile is assumed to be generated by a function expanded in an orthonormal basis (Legendre or Fourier) plus noise. The number of bases and their coefficients, are estimated with analytic computations in a fully Bayesian manner. Thus the global estimand for every gene expression trajectory is the linear combination of some number of bases whose coefficients are estimated by a posterior distribution. In addition, the BATS framework allows various types of non-Gaussian noise models.

#### BATS simulation

We reproduce one instantiation of the simulations performed in [11]; specifically, three sets of *N *= 8000 profiles, of *n *= 11 timepoints and  replicates, for *i *= 1; ..., *N*, *j *= 1, ..., *n *except  according to the model defined in [11, sec. 2.2]. In each of the three sets of profiles, 600 out of 8000 are randomly chosen to be differentially expressed (labeled as "1" in the ground truth) and simulated as a sum of an orthonormal basis of Legendre polynomials with additive i.i.d.(identically and independently distributed) noise.

**Figure 2:**
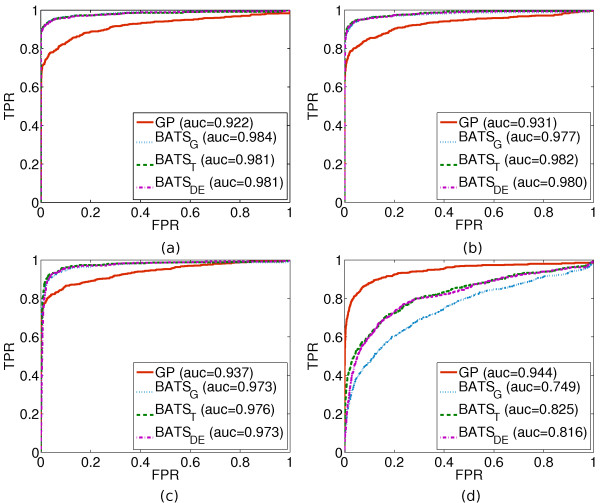
**GP vs. BATS on simulated data**. ROC curves for the GP and BATS methods on data simulated by BATS induced with **(a) **Gaussian noise, **(b) **Student's-*t *with 5 degrees of freedom, (c) Student's-*t *with 3 degrees of freedom; and on **(d) **data simulated by Gaussian processes. Each panel depicts one ROC curve for the GP method and three for BATS, each using a different noise model indicated by the subscript in the legend ("G" for Gaussian, "T" for Student's-*t *and "DE" for double exponential marginal distributions of error), followed by the area under the corresponding curve (AUC).

The other 7400 non-differentially expressed profiles (labeled as "0" in the ground truth) are essentially zero functions with additive i.i.d. noise. The three simulated datasets are induced with different kinds of i.i.d. noise; respectively, Gaussian N(0, *σ *^2^), Student-*t *distributed with 5(T(5)) and 3 (T(3)) degrees of freedom. Figure 2(a, b, c) illustrates the comparison on the BATS-sampled data with all three kinds of induced noise.

#### GP simulation

In a similar setup, the second in-silico dataset consists of 8000 profiles sampled from Gaussian processes, with the same number of replicates and time-points, among which 600 were setup as differentially expressed. To generate a differentially expressed profile, each of the *hyperparameters *of the RBF covariance function, namely the *characteristic lengthscale, signal variance *and *noise variance *(cf. **Methods**) is sampled from separate Gamma distributions. The three Gamma distributions are fitted to sets of their corresponding hyperparameters, which are observed for the true positive profiles under a near zero FPR during the first test on BATS-generated profiles. In this way, we attempt to resemble the behaviour of the BATS-sampled profiles. Table 1 lists the parameters of the three fitted Gamma distributions.

**Table 1:** Parameters of the Gamma distributions for sampling the RBF-hyperparameters.

		Sampling Gamma distribution *Γ*(*a*, *b*)	
		
		*a *(scale)	*b *(shape)
Sampled RBF- Hyperparameters	ℓ^2 ^(characteristic lengthscale)	1.4	5.7
	
	(signal variance)	2.76	0.2
	
	(noise variance)	23	0.008

These are the parameters of the Gamma distributions from which we sample the RBF- hyperparameters. For example, the characteristic lengthscale is sampled from a Gamma with scale 1.4 and shape 5.7. The hyperparameters are then used in the RBF covariance function to sample/simulate a profile from the Gaussian process.

The other 7400 non-differentially expressed profiles are simply zero functions with additive white Gaussian noise of variance equal to the sum of two samples from the Gamma distribution for the *signal variance *and the *noise variance*. This addition serves to create a non-differentiated profile of comparative scale to the differentiated ones, but nonetheless of completely random nature. Figure 2(d) illustrates the comparison on the GP-sampled data.

### Experimental data

We apply the standard GP regression framework and BATS on an experimental dataset coming from a study on primary mouse keratinocytes with an induced activation of the TRP63 transcription factor (GEO-accession number [GEOdataset:GSE10562]), where a reverse-engineering algorithm was developed (TSNI: time-series network identification) to infer the direct targets of TRP63 [13]. In that study, 786 out of 22690 gene reporters were chosen based on the area under their curves, and ranked by TSNI according to the probability of belonging to direct targets of TRP63. The ranking list was published in a supplementary file available for download

(genome.cshlp.org/content/suppl/2008/05/05/gr.073601.107.DC1/DellaGatta_SupTable1.xls) and used here as a *noisy ground truth*. We pre-process the data with the robust multi-array average (RMA) expression measure [30], implemented in the "affy" R-package.

**Figure 3:**
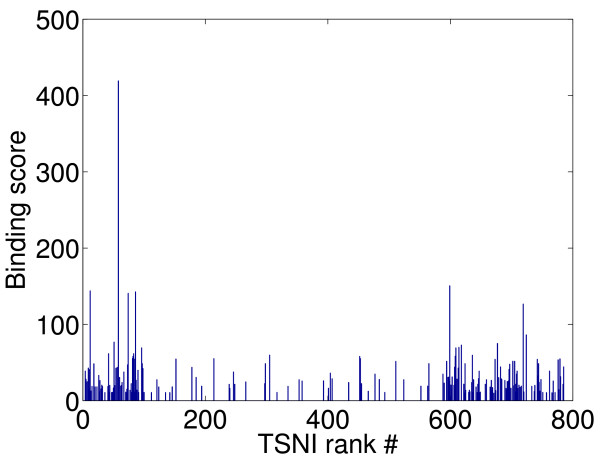
**Distribution of binding scores along the TSNI ranking**. By inspection, the distribution of the binding scores is mostly dense along the first 100 positions of the TSNI ranking. The authors in [13] only selected the top 100 genes and the bottom 200 genes to search for binding sites and thus showed that the top 100 genes have more binding sites than the bottom 200 genes.

**Figure 4:**
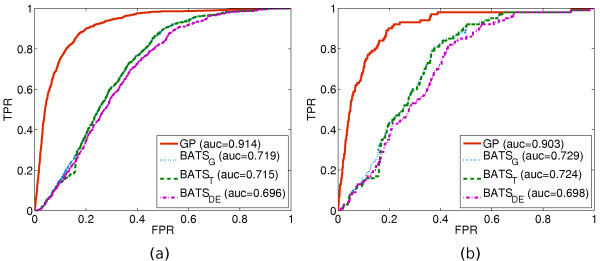
**GP vs. BATS on experimental data**. ROC curves for the GP and BATS methods on experimental data from [13]. As in Figure 2, one ROC curve and the area under it (AUC) are depicted for the GP method and three for BATS, each using a different noise model indicated by the subscript in the legend. **(a) **Ground truth consists of 22690 labels among which only the 786 profiles chosen to be ranked by TSNI (based on the area under their curves) are labeled as "1", cf. **Experimental data**. **(b) **Same number of labels; here only the top 100 profiles ranked by TSNI are labeled as "1".

We label the top 100 position of the TSNI ranking as "1" in the ground truth as they are the most likely to be direct targets of the TRP63 transcription factor and because the *binding scores *(computed as the sum of -log2 of *p*-values of all TRP63-binding regions identified by ChIP-chip experiments) are most densely distributed amongst the first 100 positions, see Figure 3. Furthermore, in [13] these 100 positions were further validated by gene set enrichment analysis (GSEA) [31] to check if their up/down regulation patterns were correlated to genes that respond to TRP63 knock-downs in general. In summary, *"the top 100 TSNI ranked transcripts are significantly enriched for the strongest binding sites" *[13]. Figure 4 illustrates the comparison on the experimental data.

## Discussion

On BATS-sampled data, Figure 2(a, b, c), we observe that the change in the induced noise is barely noticeable in regards to the performances of both methods and that BATS maintains its stable supremacy over the GP framework. This performance gap is partially due to the lack of a robust noise model for the GP (cf. **Conclusions**). Furthermore, there is a modeling bias in the underlying functions of the simulated profiles, which contain a finite small degree of differentiability (maximum degree of Legendre polynomial is 6). This puts the GP in a disadvantaged position as it models for (smooth) infinitely differentiable functions when its covariance function is a *squared exponential*. Consequently, for this simulated dataset the GP is more susceptible to capturing spurious patterns as they are more likely to lie within its modeling range, whereas for BATS modeling the polynomials with a limited degree acts as a safeguard against spurious patterns, most of which vary rapidly in time.

On GP-sampled data, Figure 2(d), we observe the reversal of the performance gap in favor of the GP framework while its performance is almost unaffected. The GP is still prone to non-differentially expressed profiles with spurious patterns and differentially expressed profiles with excessive noise. However, the limited polynomial degree of BATS proves to be inadequate for many of the GP-sampled functions and the two BATS variants with robust noise models (BATS*_T_*, BATS*_DE_*) only alleviate the problem slightly. In Figure 4 we observe the GP outperforming the Gaussian noise variant of BATS (BATS*_G_*) by a similar degree as in Figure 2(d). The experimental data are much more complex and apparently the robust BATS variants now offer no increase in performance. Since the ground truth focuses on the 100 most differentially expressed genes with respect to the induction of the TRP63 transcription factor, then these results indicate that the GP method of ranking presented here indeed highlights differentially expressed genes and that it naturally features an attractive degree of robustness against different kinds of noise.

## Conclusions

We presented an approach to estimating the continuous trajectory of gene expression time-series from microarray data through *Gaussian process *(GP) regression and ranking the differential expression of each profile via a log-ratio of marginal likelihoods of two GPs, each one representing the hypothesis of differential and non-differential expression respectively. We compared our method to a recent Bayesian hierarchical model (BATS) via ROC curves on data simulated by BATS and GPs and experimental data. Each evaluation was made on the basis of matched percentages to a ground truth - a binary vector which labeled the profiles in a dataset as differentially expressed or not. The experimental data were taken from a previous study on primary mouse keratinocytes and the top 100 genes of its ranking were used here as the noisy ground truth for the purposes of assessment. The GP framework significantly outperformed BATS on experimental and GP-sampled data and the results showed that standard GP regression can be regarded as a serious competitor in evaluating the continuous trajectories of gene expression and ranking its differential expression.

This ranking scheme presented here is reminiscent of the work in [19] on *two-sample *data (separate time-course profiles for each treatment), where the two competing hypotheses are represented in a graphical model of two different generative models connected with a *gating *scheme; one where the two profiles of the gene reporter are assumed to be generated by two different GPs, and thus the gene is *differentially expressed *across the two treatments, and one where the two profiles are assumed to be generated by the same GP, and thus the gene is *non-differentially expressed*. The gating network serves to *switch *between the two generative models, in time, to detect *intervals *of differential expression and thus allow biologists to draw conclusions about the propagation of a perturbation in a gene regulatory network. Instead, the issue presented in this paper is more basic and so is the methodology to deal with it. However, we note that the robust mechanisms against outliers used in [19,28,29] are complementary to this work and one should not hesitate to incorporate one into a framework similar to ours. Practicalities aside, this paper also introduces additional proof that Gaussian processes, naturally and without much engineering, fit to the analysis of gene expression time-series and that simplicity can still be preferred over the ever-increasing -- but sometimes necessary -- complexity of hierarchical Bayesian frameworks.

### Future work

A natural next step would be to add a robust noise mechanism in our framework. In this regard, fine examples can be found in [19,28,29]. Finally, an interesting biological question is about the potential periodicity of the underlying signal in a gene expression profile. In this regard a different of kind stationary covariance function, the *periodic *covariance function [22], can fit a time-series generated by an periodic process and thus its lengthscale hyperparameter can be interpreted as its cycle.

## Methods

As we mentioned earlier, analysing time-course microarray data by means of Gaussian process (GP) regression is not a new idea (cf. **Background**). In this section we review the methodology to estimating the continuous trajectory of a gene expression by GP regression and subsequently describe a likelihood-ratio approach to ranking the differential expression of its profile. The following content is based on the key components of GP theory as described in [21,22].

### The Gaussian process model

The idea is to treat trajectory estimation given the observations (gene expression time-series) as an interpolation problem on functions of one dimension. By assuming the observations have Gaussian-distributed noise, the computations for prediction become tractable and involve only the manipulation of linear algebra rules.

#### A finite parametric model

We begin the derivation of the GP regression model by defining a standard *linear regression *model (a more concrete example of such a model is for *ϕ *= (1, *x*, *x*^2^)^⊤^, i.e. a line mapped to a quadratic curve)(1)

where gene expression measurements in time **y **= {*y_n_*}_*n *= 1..*N *_are contaminated with white Gaussian noise and the inputs (of time) are mapped to a feature space **Φ **= {*ϕ *(*x_n_*)^⊤^}_*n *= 1..*N*_. Furthermore, if we assume the noise to be i.i.d. (identically and independently distributed) as a Gaussian with zero mean and variance (2)

then the probability density of the observations given the inputs and parameters (*data likelihood*) is Gaussian-distributed(3)

Where .

#### Introducing Bayesian methodology

Now turning to *Bayesian linear regression*, we wish to encode our initial beliefs about the parameters **w **by specifying a zero mean, isotropic Gaussian distribution as a *prior *over the parameters(4)

By integrating the product of the *likelihood × prior *with respect to the parameters, we get the *marginal likelihood*(5)

which is jointly Gaussian. Hence the *mean *and *covariance *of the *marginal *are(6)(7)(8)

By computing the *marginal likelihood *in eq. (8), we can *compare *or *rank *different models, without fear of *overfitting *on the data, or having to explicitly apply a *regulariser *to the *likelihood; *the *marginal likelihood *implicitly *penalises *too complex models [21, sec. 5.4].

Notice in eq. (7) how the structure of the covariance implies that choosing a different feature space Φ results in a different **K***_y_*. Whatever **K***_y _*is, it must satisfy the following requirements to be a valid covariance matrix of the GP:

• **Kolmogorov consistency**, which is satisfied when *K_ij _*= *K*(*x_i_*, *x_j_*) for some *covariance function K*, such that all possible **K **are *positive semidefinite *(**y^⊤ ^Ky **≥ 0).

• **Exchangeability**, which is satisfied when the data are i.i.d.. It means that the order in which the data become available has no impact on the *marginal distribution*, hence there is no need to hold out data from the training set for *validation *purposes (for measuring generalisation errors, etc.).

#### Definition of a Gaussian process

More formally, *a Gaussian process is a stochastic process (or collection of random variables) over a feature space, such that the distribution p *(*y*(*x*_1_*)*, *y*(*x*_2_),..., *y(x_n_*)) *of a function y(x), for any finite set of points *{*x*_1_, *x*_2_, ..., *x_n_*} *mapped to that space, is Gaussian, and such that any of these Gaussian distributions is Kolmogorov consistent*.

If we remove the *noise term ***I **from **K***_y _*in eq. (7) we can have noiseless predictions of *f*(*x*) rather than *y*(*x*) = *f*(*x*) + ε. However, when dealing with finite parameter spaces **K***_f _*may be *ill-conditioned *(cf. sec. *SE derivation*), so the *noise term *guarantees that **K***_y _*will have *full rank *(and an inverse). Having said that, we now formulate the GP *prior *over the *latent *function values *f *by rewriting eq. (8) as(9)

where the *mean function *(usually defined as the zero function) and the *covariance function *respectively are(10)(11)

### The squared-exponential kernel

In this paper we only use the univariate version of the squared-exponential (SE) kernel. But before embarking on its analysis, the reader should be aware of the existing wide variety of kernel families, and potential combinations of them. A comprehensive review of the literature on covariance functions is found in [21, chap. 4].

#### Derivation and interpretation of the SE kernel

In the GP definition section we mentioned the possibility of an *ill-conditioned *covariance matrix. In the case of a finite parametric model (as in eq. (1)), **K_f _**can have at most as many non-zero eigenvalues as the number of parameters in the model. Hence for any problem of any given size, the matrix is non-invertible. Ensuring **K***_f _*is not ill-conditioned, involves adding the diagonal noise term to the covariance. In an infinite-dimensional feature space, one would not have to worry about this issue as the features are integrated out and the covariance between datapoints is no longer expressed in terms of the features but by a *covariance function*. As demonstrated in [22, sec.45.3] and [21, sec.4.2.1], with an example of a one-dimensional dataset, we express the covariance matrix **K***_f _*in terms of the features **Φ**(12)

then by considering a feature space defined by *radial basis functions *and integrating with respect to their centers *h*, eq. (12) becomes(13)

where one ends up with a smooth (infinitely differentiable) function on an infinite-dimensional space of (radial basis function) features. Taking the constant out front as a *signal variance * and squaring the exponential gives rise to the standard form of the *univariate squared-exponential *(SE) covariance function. The *noisy univariate *SE kernel -- the one used in this paper is(14)

**Figure 5:**
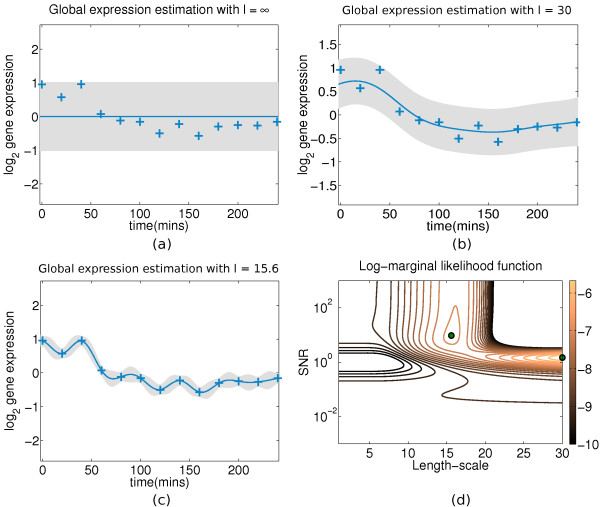
**Gaussian process fit on expression profile of gene Cyp1b1 in the experimental mouse data**. Figure 5: A GP fitted on the centred profile of the gene *Cyp1b1 *(probeID 1416612_at in the *GSE10562 *dataset) with different settings of the lengthscale hyperparameter ℓ^2^. The blue crosses represent zero-mean hybridised gene expression in time (log2 ratios between treatment and control) and the shaded area indicates the point-wise mean plus/minus two times the standard deviation (95% confidence region). **(a) **Mean function is constant as ℓ^2 ^→ ∞ (0 inverse lengthscale in eq. (14)) and all of the observed data variance is attributed to noise (). **(b) **The lengthscale is manually set to a local-optimum large value (ℓ^2 ^= 30) and thus the mean function roughly fits the data-points. The observed data variance is equally attributed to signal () and noise (). Consequently, the GP features high uncertainty in its predictive curve. **(c) **The lengthscale is manually set to a local-optimum small value (ℓ^2 ^= 15.6) and thus the mean function tighly fits the data-points with high certainty. The interpretation from the covariance function in this case is that the profile contains a minimal amount of noise and that most of the observed data variance is attributed to the underlying signal (). **(d) **The contour of the corresponding LML function plotted by an exhaustive search of ℓ^2 ^and SNR values. The two main local-optima are indicated by the green dots and a third optimum that corresponds to the 1st panel appears almost as flat in the contour and its vicinity encompasses the whole lengthscale axis for very small values of SNR (i.e. given that SNR ≈ 0, the lengthscale is trivial).

The SE is a *stationary *kernel, i.e. it is a function of *d *= *x_i _*- *x_j _*which makes it *translation invariant *in time. *δ_ij _*is the *Kronecker delta *function which is unity when *i *= *j *and zero otherwise and *l*^2 ^is the *characteristic lengthscale *which specifies the distance beyond which any two inputs (*x_i_*, *x_j_*) become uncorrelated. In effect, the lengthscale *l*^2 ^governs the amount that *f *varies along the input (time). A small lengthscale means that *f *varies rapidly along time, and a very large lengthscale means that *f *behaves almost as a constant function, see Figure 5. This parameterisation of the SE kernel becomes very powerful when combined with *hyperparameter adaptation*, as described in a following section. Other adapted hyperparameters include the *signal variance * which is a vertical scale of function variation and the *noise variance * (introduced in eq. (2)) which is not a hyperparameter of the SE itself, but unless we consider it as a constant in the *noisy *case, its adaptation can give different explanations about the latent function that generates the data.

One can also combine covariance functions as long as they are *positive-definite*. Examples of valid combined covariance functions include the *sum *and *convolution *of two covariance functions. In fact, eq. (14) is a combined sum of the SE kernel with the covariance function of *isotropic Gaussian noise*.

### Gaussian process prediction

To interpolate the trajectory of gene expression at non-sampled time-points, as illustrated in Figure 5, we infer a function value f_* _at a new input (non-sampled time-point) x_*_, given the knowledge of function estimates **f **at known time-points **x**. The joint distribution *p*(f_*_, **f **) is Gaussian, hence the conditional distribution *p*(f_*_| **f **) is also Gaussian. In this section we consider predictions using noisy observations; we know the noise is Gaussian too, so the noisy conditional distribution does not differ. By Bayes' rule(15)

where the Gaussian process prior over the noisy observations is(16)

#### Predictive equations for GP regression

We start by defining the *mean function *and the *covariance *between a new time-point x_* _and each of the *i^th ^*known time-points, where *i *= 1..*N*(17)(18)

For every new time-point a new vector k_* _is *concatenated *as an additional row and column to the covariance matrix **K***_C _*to give(19)

where C = N..N_* _is incremented with every new k_* _added to **K***_C_*. By considering a zero mean function and eq. (19), the joint distribution *p*(f*, y) from eq. (15) can be computed(20)

Finally, to derive the *predictive mean *and *covariance *of the posterior distribution from eq. (15) we use the Gaussian identities presented in [21, sec.A.2]. These are the predictive equations for *GP regression *of a single new time-point(21)(22)(23)

and *K_f _*= *K_f _*(**x**, **x**). These equations can be generalised easily for the prediction of function values at multiple new time-points by augmenting k_* _with more columns and *k*(x*, x*) with more components, one for each new time-point x*.

### Hyperparameter learning

Given the SE covariance function, one can learn the hyperparameters from the data by optimising the log-marginal likelihood function of the GP. In general, a non-parametric model such as the GP can employ a variety of kernel families whose hyperparameters can be adapted with respect to the underlying intensity and frequency of the local signal structure, and interpolate it in a probabilistic fashion (i.e. while quantifying the uncertainty of prediction). The SE kernel allows one to give intuitive interpretations of the adapted hyperparameters, especially for one-dimensional data such as a gene expression time-series, see Figure 5 for interpretations of various local-optima.

#### Optimising the marginal likelihood

In the context of GP models the marginal likelihood results from the marginalisation over function values **f**(24)

where the *GP prior p*(**f|x**) is given in eq. (9) and the likelihood is a factorised Gaussian . The integral can be evaluated analytically [21, sec. A.2] to give the *log-marginal likelihood *(LML, it is common practice to take the log of the antiderivative for the sake of numerical stability, as it yields a sum instead of a product)(25)

We notice that the marginal here is explicitly conditioned on *θ *(*hyperparameters*) to emphasise that it is a function of the hyperparameters through **K***_f_*. To optimise the marginal likelihood we take the partial derivatives of the LML with respect to the hyperparameters(26)

We use *scaled conjugate gradients *[32] -- a standard optimisation scheme -- to maximise the LML.

### Ranking with likelihood-ratios

Alternatively, one may choose to go "fully Bayesian" by placing a *hyper-prior *over the hyperparameters *p*(***θ ***|), where  represents some type of model, and compute a posterior over hyperparameters(27)

based on some initial beliefs, such as the functions having large lengthscales, and optimise the marginal likelihood so that the optimum lengthscale tends to a large value, unless there is evidence to the contrary. Depending on the model , the integral in eq. (27) may be analytically intractable and thus one has to resort to approximating this quantity [33] (e.g. Laplace approximation) or using *Markov Chain Monte Carlo *(MCMC) methods to sample from the posterior distribution [34].

In the case where one is using different types of models (e.g. with different number of hyperparameters), a Bayesian-standard way of comparing between such two models is through Bayes factors [11,19,23] -- ratios of the *integral *quantities in eq. (27)(28)

where the models  usually represent two different hypotheses, namely  - the profile has a significant underlying signal and thus it is truly differentially expressed and  - there is no underlying signal in the profile and the observed gene expression is just the effect of random noise. The ranking is based on how likely  in comparison to , given a profile.

In this paper we present a much simpler -- but effective to the task -- approach to ranking the differential expression of a profile. Instead of integrating out the hyperparameters, we approximate the Bayes factor with a log-ratio of marginal likelihoods (cf. eq. (25))(29)

with each LML being a function of different instantiations of ***θ***. We still maintain hypotheses  and  that represent the same notions explained above, but in our case they differ simply by configurations of ***θ***. Specifically, on  the hyperparameters are **fixed **to ***θ***_1 _= (∞, 0; var(**y**))^⊤ ^to encode a function constant in time (*l *^2 ^→ ∞), with no underlying signal , which generates a time-series with a variance that can be solely explained by noise . Analogously, on  the hyperparameters ***θ***_2 _are initialised to encode a function that fluctuates in accordance to a typical significant profile (e.g. ℓ^2 ^= 20), with a distinct signal variance that solely explains the observed time-series variance  and with no noise .

#### Local optima of the log-marginal likelihood (LML) function

These two configurations correspond to two points in the three-dimensional function that is the LML, both of which usually lie close to local-optimum solutions. This assumption can be verified, empirically, by exhaustively plotting the LML function for a number of profiles, see Figure 5. In case the LML contour differs for some profiles, more initialisation points should be used to ensure convergence to the maximum-likelihood solution. Because the configuration of the second hypothesis (no noise, ) is an extremely unlikely scenario, we let ***θ***_2 _adapt to a given profile by optimising the LML function, as opposed to keeping it fixed like ***θ***_1_.

In most cases the LML (eq. (25)) is not convex. Multiple optima do not necessarily pose a threat here; depending on the data and as long as they have similar function values, multiple optima present alternative interpretations on the observations. To alleviate the problem of spurious local optimum solutions however, we make the following observation: when we explicitly restrict the signal variance hyperparameter () to low values during optimisation, we also implicitly restrict the noise variance hyperparameter () to large values. This occurs as the explanation of the observed data variance (var(**y**)) is shared between the signal and noise variance hyperparameters, i.e. . This dependency allows us to treat the three-dimension optimisation problem as a two-dimension problem, one of lengthscale ℓ ^2 ^and one of signal-to-noise ratio  without fear of missing out an optima.

Figure 5 illustrates the marginal likelihood as a function of the characteristic lengthscale ℓ^2 ^and the SNR. It features two local optima, one for a small lengthscale and a high SNR, where the observed data are explained with a relatively complex function and a small noise variance, and one optimum for a large lengthscale and a low SNR, where the data are explained by a simpler function with high noise variance. We also notice that the first optimum has a lower LML. This relates to the algebraic structure of the LML (eq. (25)); the first term (dot product) promotes data fitness and the second term (determinant) penalizes the complexity of the model [21, sec.5.4]. Overall, the LML function of the Gaussian process offers a good fitness-complexity trade-off without the need for additional regularisation. Optionally, one can use multiple initialisation points focusing on different non-infinite lengthscales to deal with the multiple local optima along the lengthscale axis, and pick the best solution (max LML) to represent the  hypothesis in the likelihood-ratio during the ranking stage.

### Source code

The source code for the GP regression framework is available in MATLAB code http://staffwww.dcs.shef.ac.uk/people/N.Lawrence/gp/ and as a package for the R statistical computing language http://cran.r-project.org/web/packages/gptk/. The routines for the estimation and ranking of the gene expression time-series are available upon request for both languages. The time needed to analyse the 22690 profiles in the experimental data, with only the basic two initialisation points of hyperparameters, is about 30 minutes on a desktop running Ubuntu 10.04 with a dual-core CPU at 2.8 GHz and 3.2 GiB of memory.

## Authors' contributions

AAK designed and implemented the computational analysis and ranking scheme presented here, assessed the various methods and drafted the manuscript. NDL pre-processed the experimental data and wrote the original Gaussian process toolkit for MATLAB and AAK rewrote it for the R statistical language. Both AAK and NDL participated in interpreting the results and revising the manuscript. All authors read and approved the final manuscript.
